# Adult intussusceptions induced by a terminal ileum diverticulum: a case report

**DOI:** 10.1002/ccr3.1403

**Published:** 2018-02-23

**Authors:** Nobuhiro Nakazawa, Hideki Suzuki, Gen Ebara, Yu Watanabe, Ritsuko Tsukagoshi, Keisuke Ieta, Koji Morohara, Hidenobu Osawa, Kazuhisa Katayama, Yasukuni Yasuda, Shigehumi Tanaka, Hiroyuki Kuwano

**Affiliations:** ^1^ Department of Surgery Isesaki Municipal Hospital Isesaki Japan; ^2^ Department of General Surgical Science Gunma University Graduate School of Medicine Maebashi Japan

**Keywords:** Adult intussusception, jejunoileal diverticulum, surgery

## Abstract

We herein report a case of adult intussusceptions induced by a terminal ileum diverticulum. Histological examination confirmed a terminal ileum diverticulum full of feces, and it was considered an infiltrated region. The clinical characteristics of previously reported adult intussusceptions are also discussed, including jejunoileal diverticulum and surgical management.

## Introduction

An intussusception is the invagination of a bowel segment into its adjacent segment. This condition is rare in adults, with around two to three cases occurring in a population of 1,000,000 per annum, and accounts for <0.1% of all adult hospital admissions 1, 2, 3. Intussusceptions are much more common in children and are the most common cause of bowel obstruction. Most (95%) intussusceptions in children are idiopathic 4. The lead point is thought to be caused by lymphoid hyperplasia after a viral infection 5. In adults, 80–90% of intussusceptions have an identifiable etiology 6. The etiologies in the small bowel include adhesions, Meckel's diverticulum, inflammatory bowel disease, and benign tumors such as lipomas and adenomatous polyps. Malignant lesions in the small bowel account for 20–50% of all intussusceptions and are usually due to metastases from tumors such as melanoma and lymphoma 7. Malignant lesions are more common in the large bowel, and one of their main causes is colon adenocarcinoma 8.

Jejunoileal diverticulum (JID), except for Meckel's diverticulum, is very rare. The incidence of JID is estimated at 0.06–4.5% of the population, based on radiographic and autopsy data 9. Herein, we report a case of adult intussusception caused by a terminal ileum diverticulum.

To the best of our knowledge, this is the first report of an adult intussusception caused by a terminal ileum diverticulum with no inflammation. We also present a discussion of some related publications on this subject.

## Case Report

In April 2016, an 82‐year‐old Japanese man was admitted to our hospital because of a 1‐day history of intermittent abdominal pain. The patient had a past medical history of significant hypertension and type 2 diabetes. The patient had no history of colonic diverticulosis or previous surgery.

On physical examination, the patient was afebrile (auricular temperature 36.8°C). Although his blood pressure was high (170/95 mmHg), his other vital signs were stable. Abdominal examination revealed right hypochondrial pain, but without rebound tenderness.

Laboratory investigation revealed an inflammatory reaction (white cell count of 11,200 *μ*L, C‐reactive protein 0.25 mg/dL), renal dysfunction (blood urea nitrogen of 29 mg/dL, creatinine of 3.24 mg/dL), and hyperglycemia (blood sugar level of 300 mg/dL). The other biochemical test results were within the normal range.

**Figure 1:**
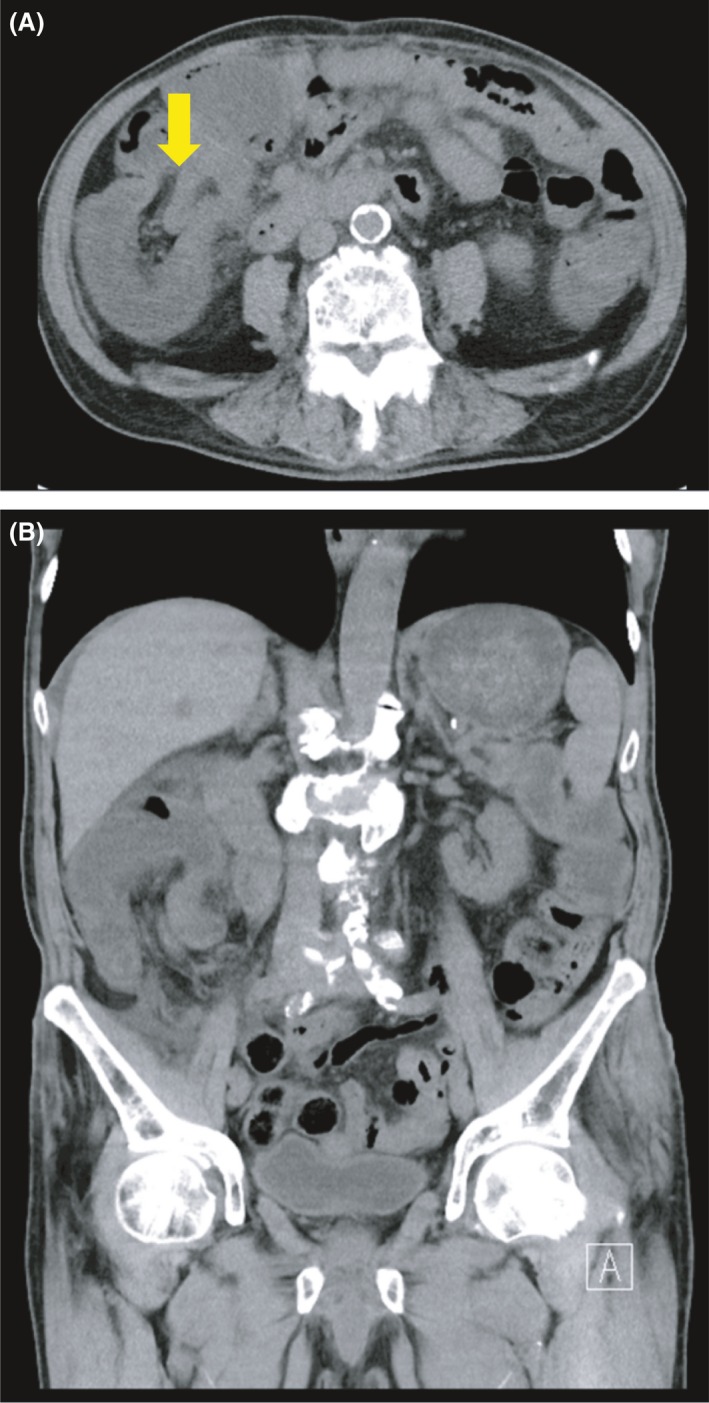
(A, B) CT finding. A plain CT scan of the abdomen and pelvis revealed a pseudokidney sign from the terminal ileum to the ascending colon.

Because of renal dysfunction, we could not plan a contrast‐enhanced CT scan. A plain CT scan of the abdomen and pelvis revealed an ileocolic intussusception (terminal ileum prolapses within the ascending colon). The axial view showed the characteristic “pseudokidney sign” in the upper right quadrant (Fig. 1A and B). Based on the physical and clinical findings, we diagnosed the patient with intussusception at the terminal ileum. For treatment, we presented both endoscopic and surgical methods to the patient. The patient chose surgical treatment for the reason recurrence prevention and possibility of malignant diseases. And the patient underwent emergency surgery.

**Figure 2:**
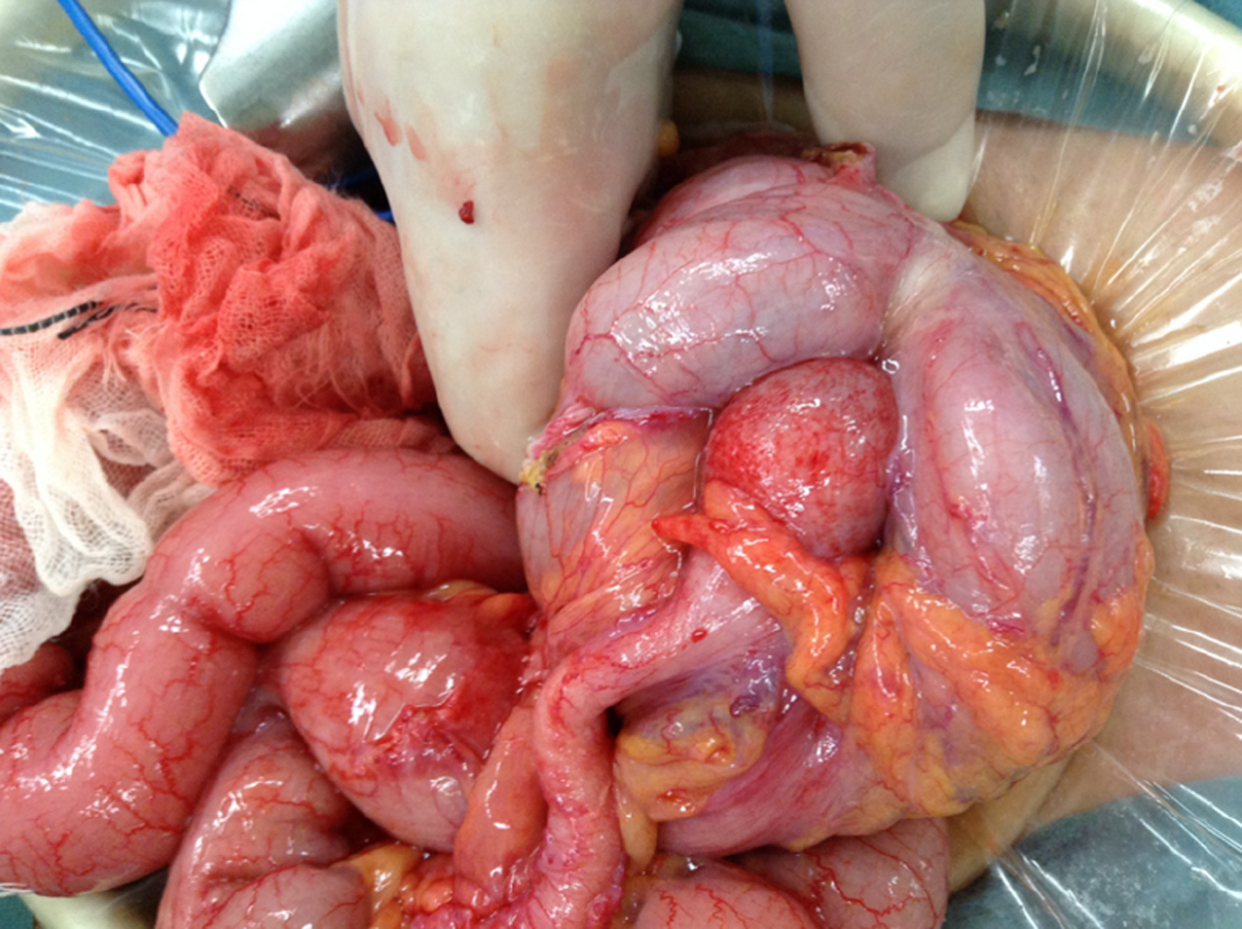
Intraoperative finding. The ileum, measuring approximately 20 cm, was invaginated at the terminal end and the ileocecal valve, and an intussusception had occurred.

After laparotomy, we found that the ileum, measuring approximately 20 cm, was invaginated at the terminal end and the ileocecal valve, and an intussusception had occurred (Fig. 2). There were small serous ascites, but the cytodiagnosis showed no malignancy. Other operative findings were that the liver surface was smooth, and there were no peritoneal nodules. Furthermore, we found no intestinal color change. We pushed the intussusception back from the distal to the proximal ileum. After reduction in the intussusception, we performed an ileocecal resection to prevent a relapse. Approximately 20 cm of intestine, including the infiltrated region, was resected, and we created an end‐to‐end anastomosis.

**Figure 3:**
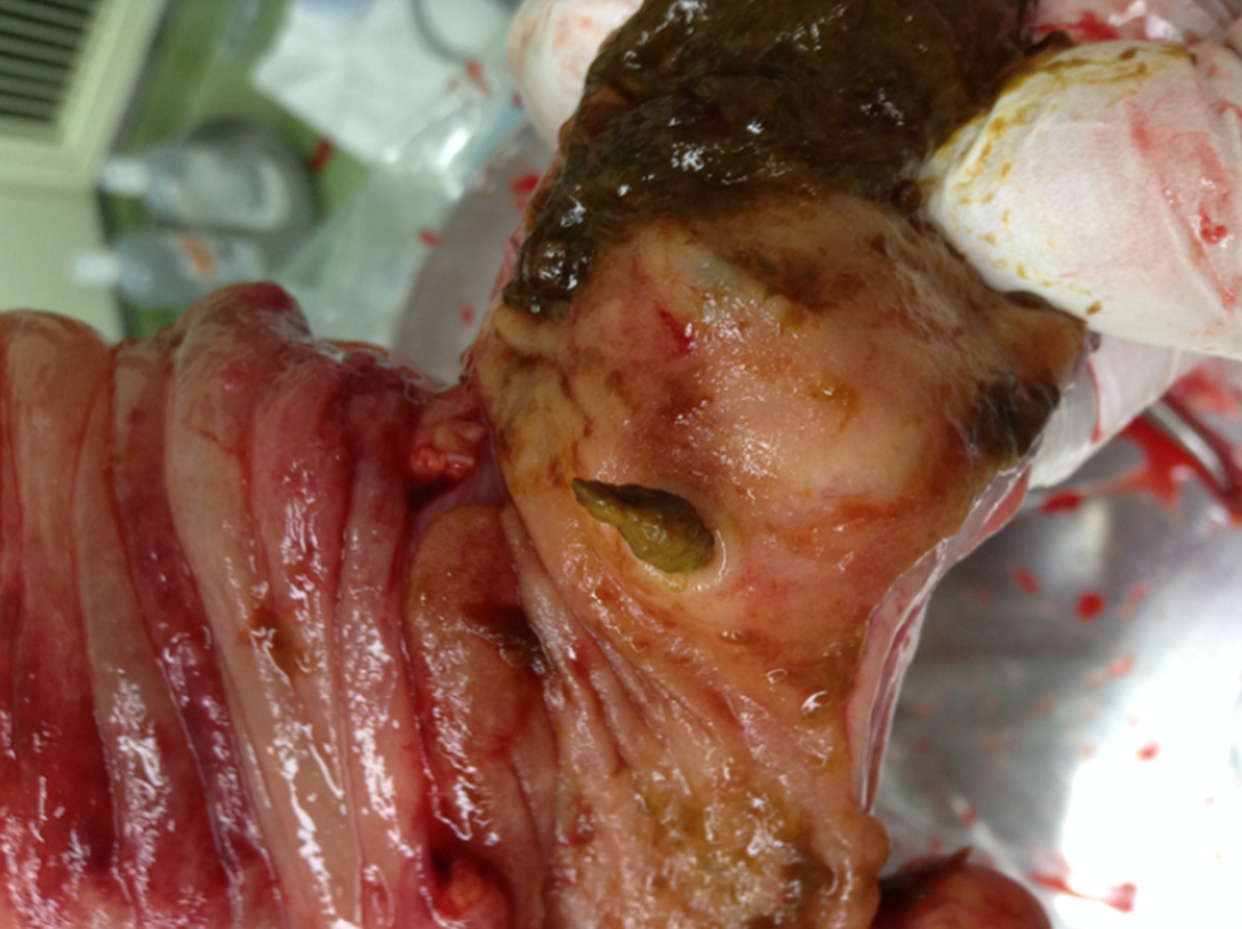
Gross pathology. There was a terminal ileum diverticulum, and the terminal ileum diverticulum was full of feces. We considered the terminal ileum diverticulum with full of feces an infiltrated region.

**Figure 4:**
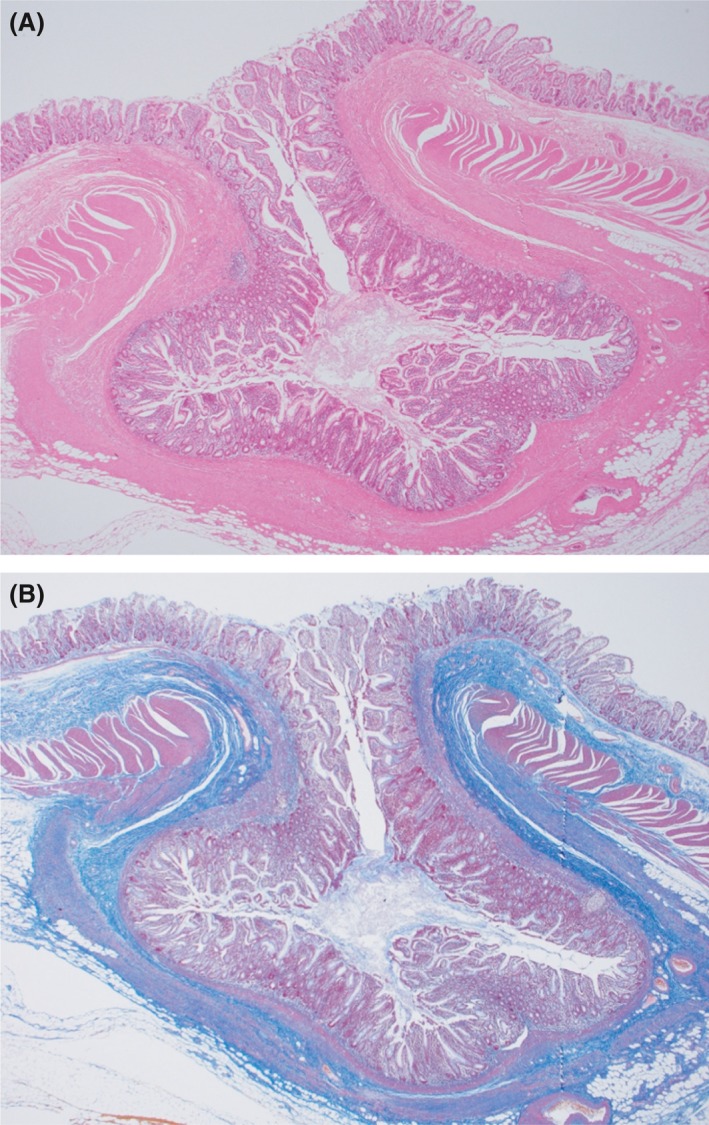
Microscopic examination. (A) Hematoxylin and eosin stain ×20. The muscular layer was absent. The pathological diagnosis was pseudodiverticulum. (B) Masson's trichrome stain ×20. There was much fibrosis at the Masson's trichrome stain.

Histological examination confirmed that the terminal ileum diverticulum was full of feces, and it was considered an infiltrated region (Fig. 3). Pathological examination confirmed that the muscular layer was absent, and there was much fibrosis at the Masson's trichrome stain (Fig. 4A and B). The pathological diagnosis was pseudodiverticulum, ileum. Meckel's diverticulum is a true diverticulum, consisting of all layers of the bowel wall (mucosa, submucosa, and muscular layer). But in our case, the muscular layer was absent and the pathological diagnosis was pseudodiverticulum. In the point, it was judged to be different from Meckel's diverticulum. The postoperative period was uneventful, and the patient was discharged on day 10.

## Discussion

Intussusceptions are rare in adults, with around two to three cases occurring in a population of 1,000,000 per annum, and account for <0.1% of all adult hospital admissions 1, 2, 3. Intussusceptions are much common in children and are the most common cause of bowel obstruction. Most (95%) intussusceptions in children are idiopathic 4. The lead point is thought to be caused by lymphoid hyperplasia after a viral infection 5. In adults, 80–90% of intussusceptions have an identifiable etiology 6. The most common classification system divides intussusceptions into four categories: enteroenteric (small bowel only), colocolic (large bowel only), ileocolic (terminal ileum prolapses within the ascending colon), and ileocecal (ileocecal valve is the lead point). Ileocolic intussusceptions are those with a prolapse of the ileum into the colon through the ileocecal valve. Thus, this case is ileocolic. The majority of intussusceptions arising in the small bowel are due to benign neoplasms, such as adhesions, Meckel's diverticulum, inflammatory bowel disease, and benign tumors. Malignant lesions in the small bowel account for 20–50% of all intussusceptions and are usually due to metastases from tumors such as melanoma and lymphoma 7. Malignant lesions are more common in the large bowel, and one of their main causes is colon adenocarcinoma 8.

In our case, the intussusception was caused by a terminal ileum diverticulum. Jejunoileal diverticulum, except for Meckel's diverticulum, is very rare. The incidence of JID is estimated at 0.06–4.5% of the population, based on radiographic and autopsy data 9. With JID, 80% of diverticula occur in the jejunum, 15% in the ileum, and 5% in both the jejunum and ileum 10. Diverticula of the small bowel occurs twice as frequently in males as in females. This prevalence increases with age, peaking when individuals are in their sixties and seventies 11. JID is thought to produce abnormalities in smooth muscle. These abnormalities could lead to distorted smooth muscle contractions. The distorted contractions could increase intraluminal pressure and cause subsequent herniation of the mucosa and submucosa through the weakest mesenteric site of the bowel wall 11, 12. JID tends to be a pseudodiverticulum (the muscular layer is absent).

A Medline search was performed using the keywords “adult intussusception” and “terminal ileum diverticulum.” No case has been reported in the English literature during the past decade. In our case, the terminal ileum diverticulum was full of faces and was hard, similar to a lipoma. Thus, it was considered an infiltrated region.

In adult intussusception, surgical resection remains the recommended treatment due to the relatively high incidence of malignancy; however, the optimal surgical management remains controversial 7, 13. Malignant lesions are more common in large‐bowel intussusceptions, so resection without reduction is recommended to avoid perforation and tumor dissemination by the intraluminal or venous route. However, in small‐bowel intussusceptions, malignancy is less frequent. For this reason, small‐bowel intussusceptions can be managed nonoperatively. The advantages of nonoperative management are that it is minimally invasive, preserves a considerable length of the bowel, and prevents the development of short bowel syndrome. In our case, considering the ileocolic type (malignancy is less frequent) and the patient's age, hypertension, severe diabetes, and clinical epidemiology, we should have highly recommended the management method of endoscopic reduction.

In conclusion, this is the first report of an adult intussusception caused by a terminal ileum diverticulum with no inflammation. The terminal ileum diverticulum was full of feces, and it was considered an infiltrated region. Our findings suggest that patients with large‐bowel intussusceptions require resection of the involved bowel without attempted reduction. However, in small‐bowel intussusceptions that are less frequently malignant. So we should have proposed a little more endoscopic reduction. On a case‐by‐case basis, we should consider nonoperative management.

## Authorship

NN: wrote the manuscript as main author. NN and HS: participated in study conception and design. NN and HS: performed operation. NN, HS, GE, YW, RT, KI, KM, HO, KK, YY, and ST: contributed treatment of the patient. HK: involved in overall supervision of the writing of this manuscript.

## Conflict of Interest

Nobuhiro Nakazawa and other coauthors have no conflict of interest. We do not have the source of support in the form of grants, equipment, and drugs.
